# Secular trend of Kawasaki disease and its correlation with viral activity in Taiwan: a nationwide population-based study

**DOI:** 10.1186/s12889-024-19066-9

**Published:** 2024-06-13

**Authors:** Wei-Liang Shih, Li-Min Huang, Mei-Hwan Wu, Luan-Yin Chang

**Affiliations:** 1 Institute of Epidemiology and Preventive Medicine, College of Public Health, National Taiwan University, No. 17, Xu-Zhou Road, Taipei, Taiwan; 2 Infectious Diseases Research and Education Center, Ministry of Health and Welfare and National Taiwan University, No. 17, Xu-Zhou Road, Taipei, Taiwan; 3 Department of Pediatrics,, National Taiwan University Hospital, College of Medicine, National Taiwan University, No. 8, Chung-Shan South Road, Taipei, Taiwan

**Keywords:** Kawasaki disease, Viral activity, Seasonality, Secular trend

## Abstract

**Background:**

Kawasaki disease (KD) is the most important acquired heart disease in children. This study investigated annual incidence, seasonality, secular trend and the correlation of KD incidence with viral activity in Taiwan.

**Methods:**

Through the national health insurance database, we identified KD during 2001–2020. The viral activity was obtained from nationwide surveillance database. We analyzed KD age-specific annual incidence, secular trends, seasonality and the correlation between KD incidence and common enteric or respiratory viral activity.

**Results:**

The KD incidence of subjects younger than 18 years significantly increased from 2001 to 2020 (11.78 and 22.40 per 100,000 person-years, respectively), and substantially decreased with age. Infants younger than 1 year presented the highest KD annual incidence at 105.82 to 164.34 per 100,000 person-years from 2001 to 2020. For all KD patients, the most frequently occurring season was summer followed by autumn. The KD incidence of infants younger than 1 year had significantly positive correlation with enteric (*r* = 0.14) and respiratory (*r* = 0.18) viral activity.

**Conclusions:**

This study demonstrates the increasing trend of KD annual incidence and seasonality (more in summer and autumn) in Taiwan. The activity of common respiratory and enteric viruses was significantly correlated with KD incidence in infants.

**Supplementary Information:**

The online version contains supplementary material available at 10.1186/s12889-024-19066-9.

## Introduction

Kawasaki disease, the most common cause of acquired heart diseases in children, is an acute vasculitis with a syndromic constellation of fever, lymphadenopathy, and mucocutaneous manifestations [1]. Since the first report approximately 60 years ago by Dr. Kawasaki in Japan [2], KD has maintained its enigmatic etiology. We found that the annual incidence of KD was 50 to 70 per 100,000 children under 5 years of age in Taiwan [3], which is much higher than in Western countries, the third highest in the world just after Japan and Korea [4, 5]. The difference in incidence in different countries or people of different races may be related to host genetic factors and/or environmental factors. KD is associated with a range of complications, the most important of which is the development of life-threatening coronary artery abnormalities. If not treated early with high-dose intravenous immunoglobulin, 1 in 5 patients with KD would develop coronary artery aneurysms; this risk is reduced 5-fold if intravenous immunoglobulin is administered within 10 days of fever onset [1]. Indeed, KD has replaced rheumatic fever as the leading cause of acquired cardiac disease in children in the developed world. Moreover, stenosis and occlusion progress over years and may result in coronary arterial disease or acute coronary syndrome later on. Both coronary artery bypass surgery and percutaneous intervention have been used to treat KD patients who develop myocardial ischemia as a consequence of coronary artery aneurysms and stenosis. Truly, KD is no longer a rare cause of acute coronary syndrome (ACS) presenting in young adults. A previous study reported that an acute myocardial infarction occurred in 0.08% (19/23,349) of KD patients from 2000 to 2010 in Taiwan, of whom one third were aged between 10 and 15 years (median, 15.7 years; range, 0.7–36.7 years), and a coronary intervention was performed by catheterization in 18 patients (all males) at a median age of 24.5 years and by surgery in 10 patients (male/female ratio, 4.0) at a median age of 21.7 years [6]. A recent study in Taiwan also reported a higher prevalence of young adult ACS associated with antecedent KD [7].

Since KD has maintained its etiology as a mystery, more investigations on its etiology will shed light on potential treatments and preventive strategies. KD is caused by multiple factors, including various heterogeneous infections [8, 9], environmental factors and specific host genetic factors [10, 11]. We hypothesize that preceding viral infections may contribute to KD in children with certain host genetic factors, so we investigated the correlation of KD incidence with viral activity, seasonality, secular trend, annual incidence and risk of coronary artery aneurysms of KD in Taiwan.

## Methods

### Data source and study subjects

This study was conducted by applying the National Health Insurance Research Database, which was derived from the systematic collection of the administrative and medical claims data of the National Health Insurance program covering almost 99% of the population since 1995 in Taiwan [12] and maintained by the Health and Welfare Data Science Center for ambulatory care expenditures by visits, inpatient expenditures by admissions, details of inpatient orders, registry for beneficiaries, and cause of death data from 2001 to 2020.

The nationwide subjects aged less than 18 years old and with complete information on sex and age during the study period of 1 January 2001 to 31 December 2020 were included as the study population. The diagnosis of KD and coronary artery anomalies (CAAs) were based on the International Classification of Diseases, Ninth Revision, Clinical Modification (ICD-9-CM) and ICD-10-CM. KD patients were defined as hospitalized patients with ICD-9-CM code of 446.1 and an ICD-10-CM code of M30.3 who received intravenous immunoglobulin (IVIG) treatment during the study period. IVIG treatments, single-dose IVIG 2 g/kg, were identified by using the prescription code (Anatomical Therapeutic Chemical [ATC] code J06BA02). CAAs were identified with the ICD-9-CM codes of 414.1 and 746.85 and ICD-10-CM codes of I25.4 and Q24.5. KD recurrence was defined as a period of > 1 month between 2 hospitalizations during the study period.

For ecological viral study, the viral isolation results for common enteric and respiratory viral activity were obtained from the Laboratory Surveillance System of Sample Detail Data, which is a nationwide laboratory surveillance database of Taiwan Centers for Disease Control (TCDC) set up to monitor viral activity in communities since 1999. This study was approved by the Institutional Review Boards of the National Taiwan University Hospital (approval number: 202006044RINA).

### Statistical analysis

The incidence rates (per 100,000 person-years) of KD were calculated by dividing the number of newly diagnosed KD patients by the corresponding person-years at risk. The person-years at risk of the study subjects were determined from their entry time of the study (1 January 2001) or the birth date for subjects born after 1 January 2001 to the KD diagnosis, death, or the end of the study (31 December 2020). To account for overdispersion features, secular trends and average annual percent change (AAPC) with a 95% confidence interval (CI) of KD incidence were evaluated by using a negative binomial regression model, which used the number of KD patients as the outcome and the natural logarithm of the corresponding person-years at risk as the offset. The calendar year was considered a continuous variable in the model and its coefficient β was used to calculate the AAPC with the formula [(exp(β)-1)]*100%. The differences in the distribution of KD cases among the four seasons were assessed with the chi-square goodness-of-fit test. Four seasons at KD diagnosis were defined as spring (February, March, April), summer (May, June, July), autumn (August, September, October), and winter (November, December, January). During the 20-year study period, the median (range) temperature was 19.7*°C* (15.2–24.5* °C*) in the spring, 27.8*°C* (24.7–29.7* °C*) in the summer, 27.4*°C* (23.5–29.4* °C*) in the autumn and 18.9*°C* (14.8–23.6* °C*) in the winter. There was no significant temperature difference between the first 10 years and the second 10 years. The correlation between KD incidence and common enteric or respiratory viral activity from the ecological viral study was determined by using Spearman correlation coefficient (r). Viral activity was defined as the number of viral isolations. The risk of CAAs was calculated by dividing the number of KD patients with CAAs by the number of all KD patients. All statistical analyses were conducted using the SAS statistical package (version 9.4; SAS Institute Cary, NC, USA) and a *p* value less than 0.05 was considered significant.

## Results

From 2001 to 2020, 11,446,632 subjects aged less than 18 years were enrolled as the study population. Among the study population, 15,033 KD patients were identified. The mean age (± SD) of the diagnosis for all KD patients was 1.53 ± 1.53 years (median: 1.0 year), and the male/female ratio of KD patients was 1.55. The proportion of KD recurrence was 1.5%.

### Secular trend of KD incidence

**Fig. 1:**
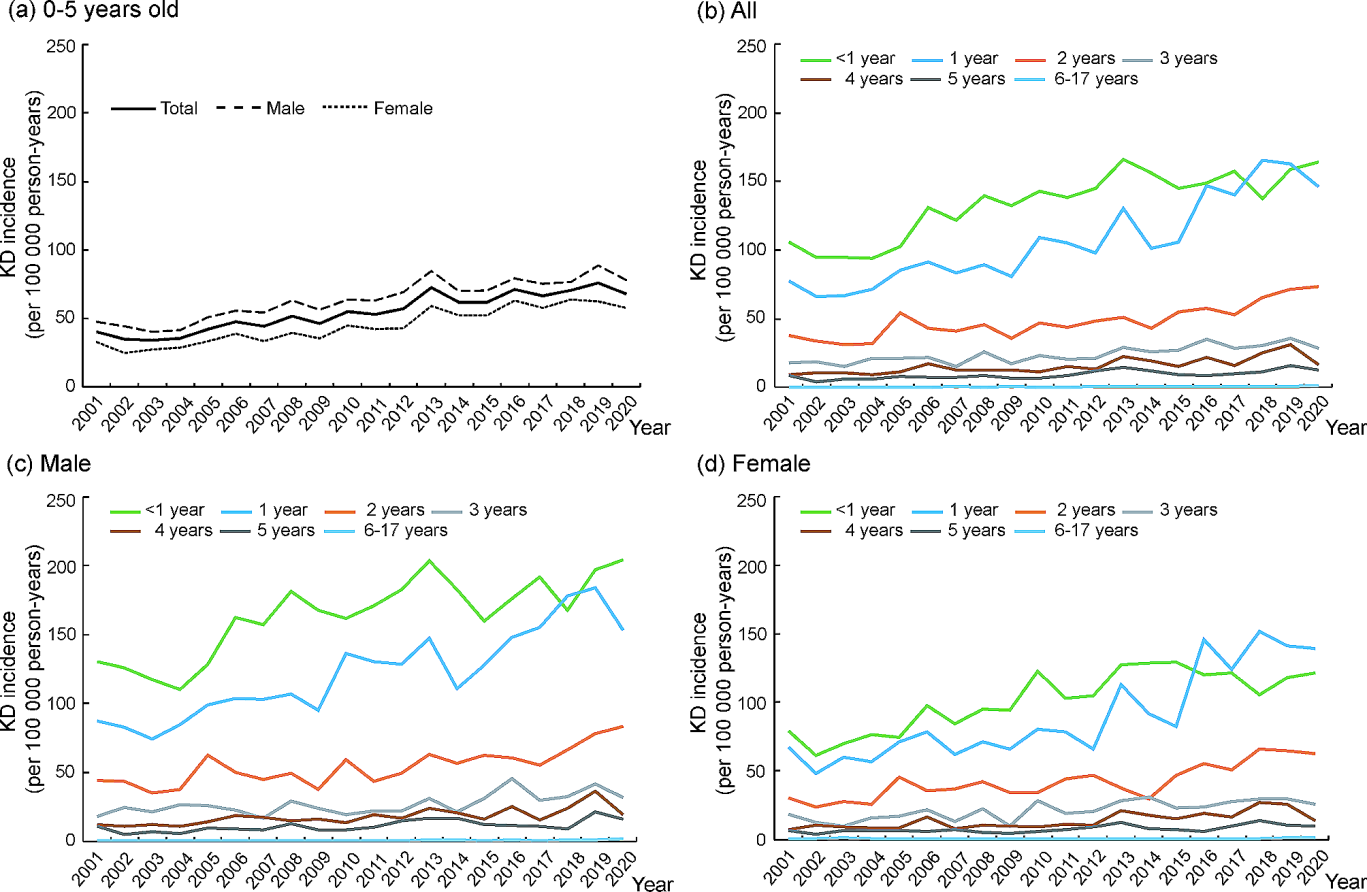
Secular trend of KD incidence in Taiwan by gender and different age groups during 2001–2020

The KD incidence of subjects younger than 18 years significantly increased from 11.78 per 100,000 person-years in 2001 to 22.40 per 100,000 person-years in 2020 (AAPC = 5.4%, *p* < 0.001, Table 1), and 97.1% of KD patients were aged 0–5 years, which also demonstrated an increasing trend with an AAPC of 4.2% (*p* < 0.001, Table 1; Fig. 1a). The KD incidence of subjects younger than 6 years ranged from 34.03 per 100,000 person-years to 75.83 per 100,000 person-years in 2001–2020, and the average annual incidence was 54.42 per 100,000 person-years. Figure 1b illustrates the age-specific KD incidence and secular trends from 2001 to 2020.

**Table 1:** Trend analysis of the KD incidence from 2001 to 2020

Age	All subjects				Male				Female		
(year)	AAPC(%)	95% CI	*p* value		AAPC(%)	95% CI	*p* value		AAPC(%)	95% CI	*p* value
< 1	2.8	(2.1 to 3.5)	< 0.001		2.6	(1.8 to 3.3)	< 0.001		3.3	(2.4 to 4.2)	< 0.001
1	4.7	(3.9 to 5.5)	< 0.001		4.2	(3.4 to 5.0)	< 0.001		5.5	(4.2 to 6.7)	< 0.001
2	3.7	(2.7 to 4.6)	< 0.001		3.2	(2.1 to 4.3)	< 0.001		4.4	(3.2 to 5.7)	< 0.001
3	3.5	(2.5 to 4.5)	< 0.001		3.0	(1.6 to 4.5)	< 0.001		4.2	(2.6 to 6.0)	< 0.001
4	4.9	(3.3 to 6.5)	< 0.001		4.3	(2.7 to 6.0)	< 0.001		5.8	(3.7 to 7.9)	< 0.001
5	4.2	(2.6 to 5.8)	< 0.001		4.3	(2.2 to 6.5)	< 0.001		3.9	(1.4 to 6.5)	0.002
6–17	4.7	(2.9 to 6.5)	< 0.001		5.7	(3.4 to 8.1)	< 0.001		3.5	(0.5 to 6.6)	0.023
0–5	4.2	(3.5 to 4.9)	< 0.001		3.8	(3.1 to 4.5)	< 0.001		4.9	(4.1 to 5.7)	< 0.001
0–17	5.4	(4.6 to 6.3)	< 0.001		4.9	(4.0 to 5.7)	< 0.001		6.2	(5.1 to 7.2)	< 0.001

AAPC: average annual percent change. CI: confidence interval

The KD incidence substantially decreased with age. Subjects aged less than 1 year presented the highest KD incidence, with an annual incidence of 105.82 to 164.34 per 100,000 person-years from 2001 to 2020. However, the KD incidence of 1-year-old subjects was equal to or even higher than that of infants younger than 1 year after 2016. The KD incidence of 2-year-old subjects was 37.58 to 73.37 100,000 person-years from 2001 to 2020. The KD incidences were less than 50 per 100,000 person-years for subjects aged 3–5 years and decreased further to approximately or lower than 1 per 100,000 person-years for subjects aged ≥ 6 years. Regardless of the difference in KD incidence rates, all age-specific KD incidences showed a statistically increased trend during the 20-year study period (all *p* < 0.001, Table 1), and the lowest change in incidence over time was observed in subjects less than 1 year (AAPC = 2.8%, Table 1).

Regarding gender analysis, the KD incidence of male subjects was higher than that of female subjects in the group of 0–5 years old during the study period (Fig. 1a). Both sexes showed that the age-specific KD incidence decreased with age (Fig. 1c and d). In addition, similar increasing and significant trend changes in age-specific KD incidences were also observed in male (*p* < 0.001 for all age groups, Table 1) and female (*p* = 0.002 for 5 years old, *p* = 0.023 for 6–17 years old and *p* < 0.001 for the other age groups, Table 1). However, the magnitude of the trend change in KD incidence was higher in females than in males from 0 to 4 years old but lower in females than in males from 5 to 17 years old (Table 1).

### Seasonality of KD onset

**Fig. 2:**
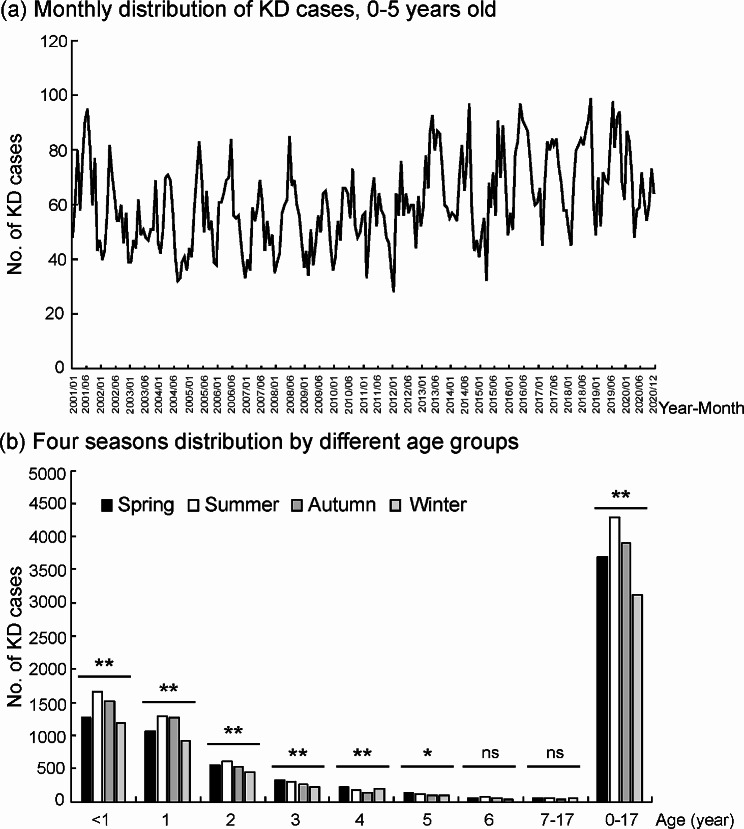
Seasonal distribution of KD cases during 2001–2020. *p value < 0.01. **p value < 0.001. ns, not significant

The monthly distribution of KD patients showed the seasonality feature of KD onset (Fig. 2a). For all KD patients, the most frequently occurring season was summer (28.6%), followed by autumn (26.0%), and the lowest incidence occurred in winter (20.8%) (*p* < 0.001). A similar pattern of seasonal distribution was observed for KD patients aged 0–2 years (all *p* < 0.001, Fig. 2b), but no significant seasonal distribution was found for KD patients aged older than 6 years.

### Correlation of KD incidence with viral activity

**Fig. 3:**
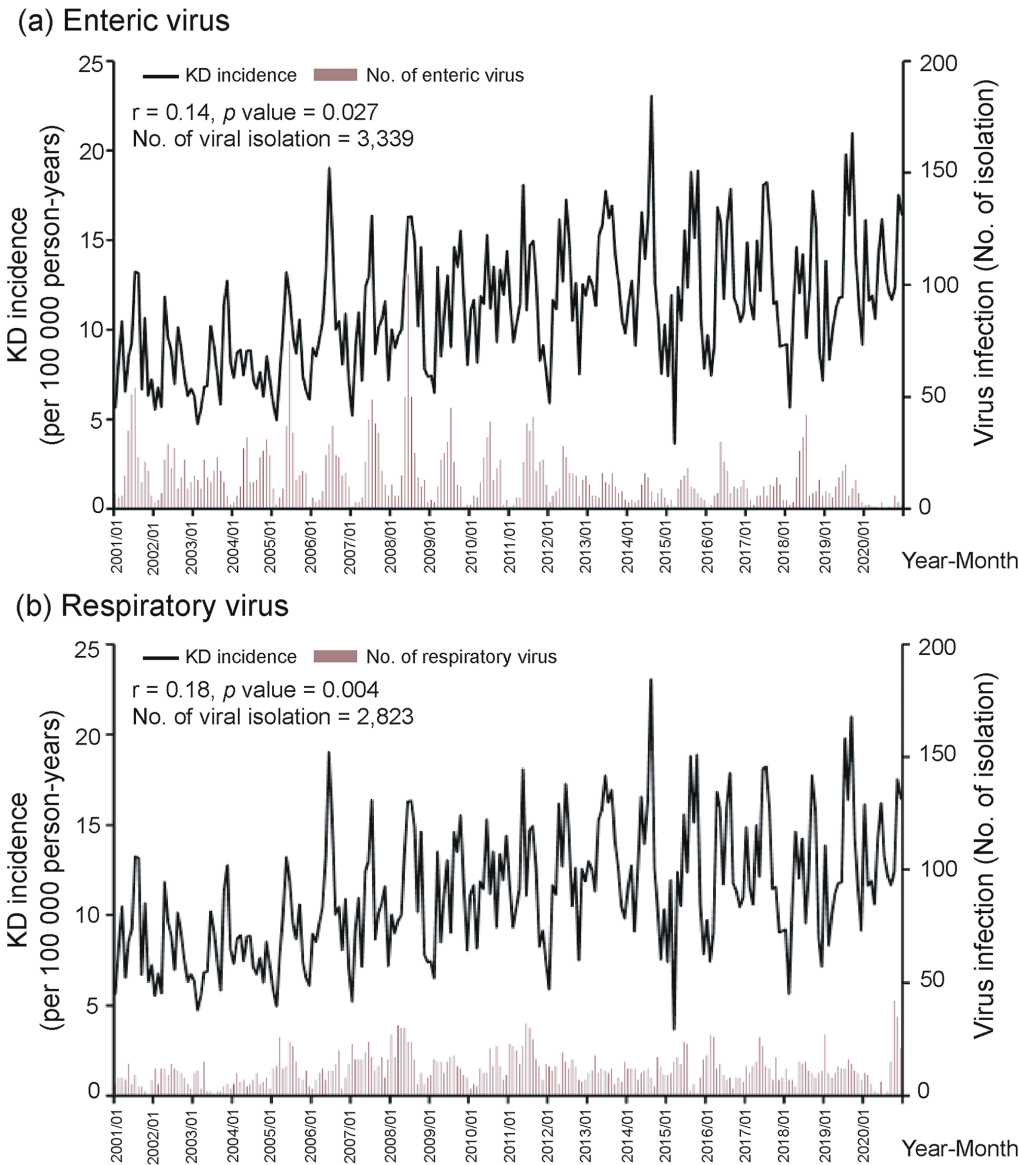
Correlation between KD incidence and viral activity for children younger than 1 year during 2001–2020

Overall, no significant correlation was observed between KD incidence and common enteric or respiratory viruses (*r* = -0.08, *p* = 0.191 and *r* = -0.08, *p* = 0.218, respectively). However, the results of subgroup analyses by age revealed that the KD incidence of patients aged less than 1 year was positively correlated with common enteric viral activity (*r* = 0.14, *p* = 0.027) and respiratory viral activity (*r* = 0.18, *p* = 0.004) (Fig. 3). The association between KD incidence and viral activity for children older than 1 year during 2001–2020 was not significant, as shown in supplementary Figure S1.

### Risk of coronary artery anomalies (CAAs)

**Fig. 4:**
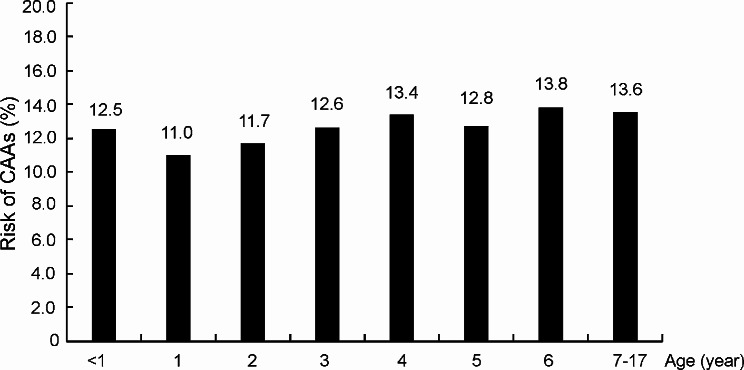
Coronary artery anomalies (CAAs) risk (%) of KD patients by different age groups during 2001–2020

Among 15,033 KD patients, the risk of the development of CAAs was 12.0%. The CAAs risk of male patients (13.1%) was significantly higher than that of female patients (10.3%) (*p* < 0.001). Figure 4 presents the age-specific CAAs risks of KD patients. The lowest CAAs risk was observed for 1-year-old KD patients. For KD patients aged over 1 year, the age-specific CAAs risks increased with age. KD patients aged less than 1 year also had a higher risk for the development of CAAs than 1-year-old KD patients.

## Discussion

Our study demonstrates the increasing trend of KD annual incidence and seasonality (more in summer and autumn) in Taiwan. For infants with the highest annual incidence, the activity of common respiratory viruses and enteric viruses was significantly correlated with KD incidence. The risk of CAAs increased with age in children older than 1 year.

The reasons why the annual incidence of KD increased with year in Taiwan need further investigation. Increased KD incidence was also observed in Japan and Beijing [4, 13]. Possible reasons include the increased awareness of physicians and early treatment. The other causes may include a low birth rate, infectious incidence and the subsequent immune response in different age groups or environmental factors such as increased air pollution. The increasing trend of infants cared at daycare centers in Taiwan may increase the infection incidence risk, which may be another possible reason for the increased KD incidence and highest KD incidence in < 1 year and 1-year-old infants. From the available data of Social and Family Affairs Administration of Ministry of Health and Welfare, the number of < 1 year infants cared at daycare center was 4,889 in 2014 and increased to 8,248 in 2020, while the number of 1-year-old infants cared at daycare center was 7,117 in 2014 and increased to 16,965 in 2020 in Taiwan. Based on the viral surveillance data from the Laboratory Surveillance System, we measured the infection incidence risk ratios among different age groups, and found the infection incidence risk ratio was highest among infants young than 1 year and 1-year-old infants, which provide the evidence of the above hypothesis.

KD incidence was highest in infants younger than 1 year, and it decreased with age. However, the incidence of 1-year-old toddlers increased and was similar to or even higher than that of infants after 2016 (Fig. 1b). With the year, the birth rate decreases, for example, the newborn population was 305, 312 in 2000 and it was only 165,249 in 2020. Moreover, the rate of a single child in one family increases in Taiwan, which may reduce the infection rate in infants younger than 1 year during recent years because no other elder sisters or brothers transmit infectious diseases to them. After they are older (possible older than 1 year) and attend daycare centers, their infection incidence will increase, as will KD incidence. Therefore, the highest incidence of KD occurred in 1-year-old children among all age groups in 2018 and 2019. This also explains why the lowest change in incidence over time was observed in infants younger than 1 year (AAPC = 2.8%, Table 1), and the increasing trend of KD annual incidence was more obvious in the other age groups.

Previous studies in different countries showed that heterogeneous viral activity was associated with KD [14–19]. Respiratory and enteric viral activity detected by routine viral isolation was correlated with KD incidence in infants younger than 1 year but not in other age groups in this study. Infants younger than 1 year were very susceptible to all kinds of viral infections after their maternal antibodies were weaned. Therefore, they might contract all kinds of viruses, their infection incidence may be higher and so is KD incidence.

KD incidence in the other age groups was not associated with identifiable viral activity in our study. Older age groups might have previous immunity against these identifiable viruses and were less susceptible. However, we believe that other viral activity, such as rhinovirus or coronavirus, undetectable by routine viral isolation [19] or other environmental factors may play more important roles in the pathogenesis of KD in older age groups [20–22].

Seasonality analysis revealed that more KD cases occurred in summer and autumn in Taiwan. It also reflected different viral activities associated with KD in different seasons. For example, enterovirus activity was usually highest in summer in Taiwan, and enterovirus was one of the most significant viruses associated with KD [18, 19]. This may explain why the peak season of KD in Taiwan also occurred in summer. The seasonality and temporal and spatial cluster differed among different areas or countries [23].

On gender analysis, the KD incidence of male subjects was higher than that of female subjects in the group of 0–5 years old during the study period, which was similar to our previous and other studies [3–5]. The causes of gender difference need further investigations. Possible causes may include different immune response, sex hormone and different infection incidence risk ratio. We measured the male to female risk ratio of infection incidence to be 1.15 based on the viral surveillance data from the Laboratory Surveillance System, which endorsed the above speculation.

CAAs rates were different in different age groups, and older age groups had a higher rate of CAAs. Male KD patients had a significantly higher risk of CAAs than female patients. This may be related to different immune reactions in different ages and sexes. Male gender was reported to have a higher rate of CAAs in other studies [24].

There are some limitations in this study. First, this is a population study with claimed data of the National Health Insurance. The accuracy of the data was not verified. Second, we could not obtain the results of the viral workup for individual KD cases. Third, the ecological viral activity obtained from the Laboratory Surveillance System of Sample Detail Data, a nationwide laboratory surveillance database of Taiwan Centers for Disease Control (TCDC), might not reflect all the viral infectious etiologies associated with KD cases. Fourth, we could not distinguish typical KD from atypical KD, incomplete KD, and KD shock syndrome based on their ICD codes.

## Conclusions

This study demonstrates the increasing trend of KD annual incidence and summer/autumn seasonality in Taiwan. The activity of common respiratory viruses and enteric viruses was significantly correlated with KD incidence in infants.

### Electronic supplementary material

Below is the link to the electronic supplementary material.

Supplementary Material 1

## Data Availability

The datasets analyzed in this study was available and applied from the Health and Welfare Data Science Center (HWDC), Ministry of Health and Welfare, Taiwan. Due to the data privacy, ethical and legal restrictions imposed by Taiwan government, the availability of the data used in this study is not publicly available. The on-site analysis and access of the raw data which were required to support the findings of this study was allowed at HWDC only. Requests for accessing the raw data can be applied as formal proposals to the HWDC (https://dep.mohw.gov.tw/DOS/cp-5283-63826-113.html).

## Abbreviations

KD: Kawasaki disease
CAAs: Coronary artery anomalies
TCDC: Taiwan Centers for Disease Control
IVIG: Intravenous immunoglobulin
AAPC: Average annual percent change
