# Supplementary figures and images for: Secular trend of Kawasaki disease and its correlation with viral activity in Taiwan: a nationwide population-based study
Source: BMC Public Health. 2024 Jun 13;24:1591. doi: 10.1186/s12889-024-19066-9 (PMC11177350; doi:10.1186/s12889-024-19066-9)

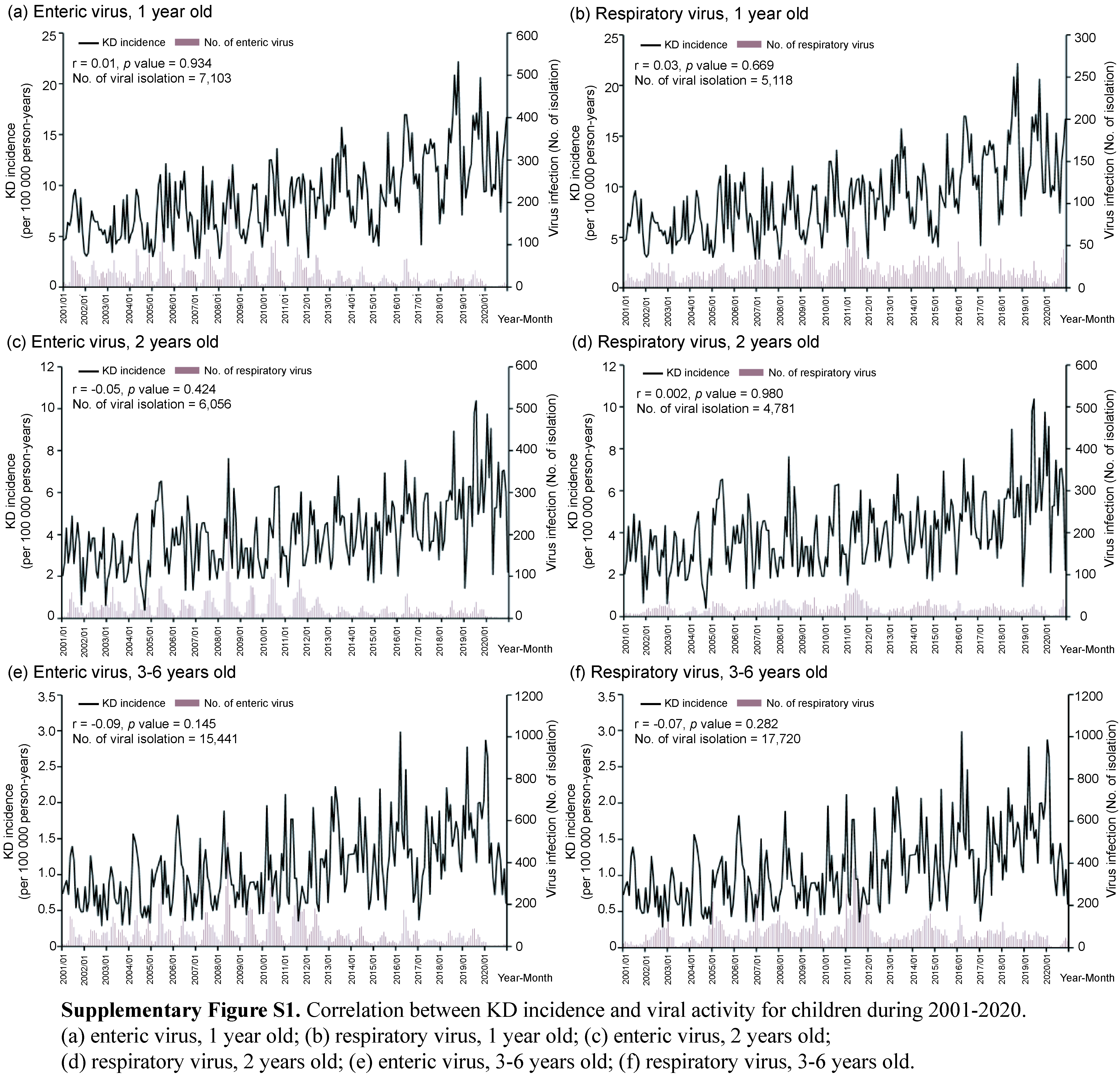

Supplement: Supplementary file 1 — Supplementary Material 1 [file 12889_2024_19066_MOESM1_ESM.tif]
